# Laptm4a mediates renal ischemia-reperfusion injury by regulating the UNC5B-AKT/mTOR signaling pathway

**DOI:** 10.3389/fimmu.2026.1683343

**Published:** 2026-03-24

**Authors:** Xiaoxiong Ma, Zhan Chen, Tianyu Wang, Zhongbao Chen, Tao Qiu, Jiangqiao Zhou

**Affiliations:** Department of Organ Transplantation, Renmin Hospital of Wuhan University, Wuhan University, Wuhan, China

**Keywords:** AKT-mTOR, apoptosis, inflammation, ischemia-reperfusion injury, laptm4a

## Abstract

**Introduction:**

Renal ischemia-reperfusion injury (IRI) is a major cause of acute kidney injury (AKI), but specific therapeutic targets are lacking.Lysosomal Protein Transmembrane 4A (LAPTM4A), a four transmembrane-spanning protein mainly localized in endosomes and lysosomes,however, its pathological role in AKI remains unexplored.

**Methods:**

To investigate the impact of LAPTM4A on renal IRI, we developed an in vivo renal IRI model utilizing LAPTM4A knockout mice, alongside an in vitro hypoxia-reperfusion(H/R) model employing shRNA to knockdown LAPTM4A in HK2 cell. A comprehensive suite of methodologies, including Western blotting, qPCR, CCK-8 assay, LDH assay, TUNEL assay, ELISA, and histopathological analysis, was employed to evaluate cell proliferation, apoptosis, and inflammatory cytokine levels.

**Results:**

We found that the expression of Laptm4a was upregulated in kidney of renal IRI mice and HK2 cell induced by hypoxia-reoxygenation(H/R). Knockout of Laptm4a improved renal function , attenuated tubular injury and reduced inflammatory cell infiltration and apoptosis, whereas overexpression exacerbated the injury.Mechanistically Laptm4a exacerbated renal IRI through suppression of the UNC5B-AKT-mTOR protective signaling pathway.

**Conclusions:**

Our findings suggest that Laptm4a exacerbates inflammation and apoptosis by inhibiting the UNC5B-PI3K-AKT signaling pathway. This study provides a novel elucidation of the role of Laptm4a as a critical positive regulator of IRI, offering preliminary preclinical insights that may inform future investigations into therapeutic strategies.

## 1 Introduction

Renal ischemia-reperfusion injury (IRI) is one of the main causes of acute kidney injury (AKI), commonly seen in clinical situations such as kidney transplantation and shock. Its pathophysiological mechanisms involve complex inflammatory responses and apoptosis processes (1). During IRI, ischemia leads to insufficient oxygen and nutrient supply to renal cells, followed by reperfusion, which increases oxidative stress. This stress can exacerbate cellular damage and death. The inflammatory response, characterized by the release of cytokines and chemokines, recruits various immune cells, further intensifying tissue injury (2, 3). Therefore, exploring the interplay between inflammatory responses and apoptosis in the context of IRI has become crucial for understanding its pathological mechanisms.

Laptm4a is a member of the lysosomal membrane protein family, which plays an important role in maintaining cellular homeostasis and responding to stress (4–6). Current research has indicated that Laptm4a is functional in vesicular transport and signal transduction in the nervous system, but its specific role in kidney diseases has not been thoroughly explored, especially concerning renal IRI, which remains a blank area of research (7). Although existing literature suggests that lysosomal membrane proteins may be involved in ischemic damage (8, 9), systematic studies on this specific member Laptm4a are still insufficient. Therefore, further investigation into the role of Laptm4a in renal ischemia will help fill this research gap and provide new directions for the treatment of AKI.

This study employs both *in vivo* and *in vitro* experiments. By constructing a mouse renal ischemia-reperfusion injury model and an HK2 cell H/R model, along with gene knockout and overexpression techniques, we systematically investigated the impact of Laptm4a on kidney injury. The research also utilized techniques such as co-immunoprecipitation (Co-IP) and immunofluorescence colocalization to explore the interaction between Laptm4a and UNC5B in depth, and rescue experiments were conducted to validate the critical role of the UNC5B-AKT/mTOR pathway. By elucidating the pathological role of Laptm4a in renal ischemia-reperfusion injury (IRI) and its molecular mechanisms, this study aims to enhance the understanding of the functions of lysosomal proteins in kidney diseases and may provide new molecular targets and intervention strategies for the clinical treatment of AKI.

## 2 Materials and methods

### 2.1 Experimental animals and model construction

Male C57BL/6j mice of 8-10 weeks of age weighing 22-30 g were selected for enrollment. Laptm4a-KO animals (global knockout) were purchased from Gempharmatech Co., Ltd All experimental mice were housed in SPF-grade animal rooms under the following conditions: room temperature of 22-24°C, humidity of 40-70%, alternating light and darkness for 12h, and free access to water and food. Animal experimental procedures were performed in accordance with the Chinese Guidelines for the Care and Use of Laboratory Animals and approved by the Ethics Committee of Wuhan University People’s Hospital.

The renal IRI model was induced by bilateral renal hilum clamping. The specific methods were as follows: mice were fasted for 6-12 hours before surgery. The mice were anesthetized by intraperitoneal injection of sodium pentobarbital. The mice were placed on a thermostatic heating pad to control their body temperature in the range of 36.5-37°C. A midline incision was made to expose the bilateral renal hilum. The renal hilum was carefully isolated, and the bilateral renal hilums were clamped with micro arterial clamps for 30 min, after which the kidneys were observed for 5 min after removal of the clamps to ensure that blood flow was restored. Tissue samples were collected 24 hours after blood re-perfusion and the mice were euthanized.The renal clitoris was exposed and not clamped in the sham-operated group. 24 hours after reperfusion, anesthetized mice were euthanized, and after thoracotomy and cardiac perfusion, the left kidney was removed and fixed in 10% formalin. The kidney was then cut into two pieces, dehydrated, embedded to prepare paraffin samples, and sectioned into 5 μm paraffin slices for subsequent staining.

### 2.2 Serum testing

After reperfusion, anesthetized mice were subjected to blood collection from the medial canthus vein. The blood was allowed to stand at room temperature for 2 hours, followed by centrifugation at 3000 g for 15 minutes to collect the serum. The serum was then diluted with physiological saline at a 1:2 ratio, and the levels of serum creatinine (CREA, Cr) and UREA nitrogen(BUN) were measured using an automated biochemical analyzer (Hitachi 3110) to evaluate kidney function.

### 2.3 Test of Tnf-α,IL-6 and IL-1β content

Mice serum concentrations of interleukin-6 (IL-6; ELK1157, ELK Biotechnology), interleukin-1 beta (IL-1β; ELK1271, ELK Biotechnology), as well as the levels of tumor necrosis factor-alpha (TNF-α; ELK1190, ELK Biotechnology), IL-6 (ELK1156, ELK Biotechnology), and IL-1β(ELK1270, ELK Biotechnology) in the HK2 cell culture medium were quantified utilizing enzyme-linked immunosorbent assay (ELISA) kits, following the manufacturer’s protocol.

### 2.4 Pathological Staining (HE, CD11B, TUNEL)

HE Staining: After deparaffinization and hydration of the paraffin sections, staining was performed using hematoxylin (G1004, Wuhan Saiweier Biotechnology Co., Ltd.) and eosin (BA-4024, Zhuhai Bess BioTechnology Co., Ltd.). Once completed, images were scanned using a slide scanner (win180, WINMEDIC). Tubular injury was assessed utilizing a double-blind scoring system predicated on the percentage of the affected area, encompassing tubular dilatation and intertubular hemorrhage. The grading scale was as follows: Grade 0 indicated no injury; Grade 1 corresponded to less than 25% affected area; Grade 2 represented 25–50% affected area; Grade 3 denoted 50–75% affected area; and Grade 4 was assigned to cases with more than 75% affected area.

CD11B Immunofluorescence: The paraffin sections were subjected to high-temperature EDTA antigen retrieval for 20 minutes, followed by blocking with 10% goat serum. The sections were then incubated overnight at 4 °C with a 1:200 dilution of CD11B antibody (BM3925, Boster). After incubation, the sections were washed with PBS, and then incubated for 1 hour with the secondary antibody (Alexa Fluor^®^ 568 goat anti-Rabbit IgG (H+L) A11036, Invitrogen, Carlsbad, CA, USA) and stained with DAPI for nuclear visualization. Observations and imaging were performed under a fluorescence microscope (OLYMPUS, BX51).

TUNEL Staining: The paraffin sections were stained using the TUNEL staining kit (Roche, 11684817910) according to the manufacturer’s instructions. DAPI was used to stain the nuclei. Observations and imaging were performed under a fluorescence microscope (OLYMPUS, BX51).

### 2.5 HK2 cell culture and induction of H/R cell model

Human renal tubular epithelial cells (HK2) were cultured in DMEM-F12 medium (PB150315-500, PunoSai) supplemented with 10% fetal bovine serum (FBS-S500, Newzerum) and 1% penicillin-streptomycin solution (PB180120, PunoSai) in a 37 °C incubator with 5% CO2. To establish a H/R model *in vitro*, the original medium was replaced with glucose-free and serum-free DMEM, and the cells were incubated for 24 hours in a culture chamber with 5% CO2, 1% O2, and 94% N2 at 37 °C. Subsequently, the glucose-free and serum-free medium was replaced with normal complete culture medium, and the cells were cultured for 6 hours in a normoxic incubator at 37 °C with 5% CO2.

### 2.6 Cell viability assessment and LDH contents detection

Cells in the logarithmic growth phase were counted and seeded into 96-well plates at a density of 5 × 10^3 cells per well, with five replicate wells for each group. Three blank wells (without cells, only with an equal volume of culture medium) were also included. After completing the H/R treatment, the CCK-8 assay kit (C0039, Beyotime) was used according to the manufacturer’s instructions, and the cells were incubated at 37 °C for 2 hours. Then, the absorbance of each group was measured at 450 nm. The LDH concentration in HK2 was measured using an LDH Cytotoxicity Assay Kit (S0026; Beyotime), according to manufacturer’s protocol.

### 2.7 Construct knockdown or overexpression stable cell lines

To establish a stable HK2 cell line overexpressing Laptm4a or UNC5B, the full-length coding sequence of the human Laptm4a or UNC5B gene was amplified from a human cDNA library and inserted into a phage vector using the ClonExpress II One-Step Cloning Kit (C112-02, Vazyme, China) to obtain the Laptm4a or UNC5B overexpression plasmid. To knock down Laptm4a, we synthesized a short hairpin RNA (shRNA) sequence targeting to the Laptm4a gene and cloned it into the pLKO.1 vector to obtain the Laptm4a knockdown plasmid. The shRNA and GFP were used as negative controls. Subsequently, the overexpression or knockdown plasmids, along with the packaging plasmids psPAX2 (1260, Addgene) and pMD2.G (12259, Addgene), were co-transfected into HEK293T cells using polyethyleneimine (PEI, 23966-1, Polysciences, USA) as the transfection reagent. After 48 hours of transfection, the harvested lentivirus was used to transfect HK2 cells. The knockdown and overexpression cell lines were obtained through continuous selection with puromycin for subsequent experiments.

### 2.8 qRT-PCR

RNA was extracted from tissues or cells using Trizol, followed by chloroform extraction to obtain RNA. The extracted RNA was then reverse transcribed into cDNA using a reverse transcription kit. Specific primers for the target genes were designed, and qRT-PCR was performed using cDNA as the template, with β-actin as the internal control, to analyze the relative expression levels of the mRNA of each gene. The primer information used is as follows in **Table 1**:

**Table 1:** Sequences of primers used for RT-qPCR.

Gene	Species	Acession #	Forward 5’--3’	Reverse 5’--3’	Amplicon size (bp)
Laptm4a	mice	NM_008640.3	GAGGTGACTCATCCAAATTCCAT	AAGAACGCAGGCATTATCAGC	102
Il6	mice	NM_031168.2	TAGTCCTTCCTACCCCAATTTCC	TTGGTCCTTAGCCACTCCTTC	76
Ccl2	mice	NM_011333.3	TACAAGAGGATCACCAGCAGC	ACCTTAGGGCAGATGCAGTT	181
Cxcl2	mice	NM_009140.2	GCGCCCAGACAGAAGTCATA	CAGTTAGCCTTGCCTTTGTTCA	118
Kim-1	mice	NM_134248.2	ATCCCATACTCCTACAGACT	CCAACATAGAAGCCCTTA	123
Ngal	mice	NM_008491.2	AAGGCAGCTTTACGATGT	TGGTTGTAGTCCGTGGTG	220
Bax	mice	NM_007527.4	TGAGCGAGTGTCTCCGGCGAAT	GCACTTTAGTGCACAGGGCCTTG	213
Bcl2	mice	NM_009741.5	TGGTGGACAACATCGCCCTGTG	GGTCGCATGCTGGGGCCATATA	118
β-actin	mice	NM_007393.5	GTGACGTTGACATCCGTAAAGA	GCCGGACTCATCGTACTCC	245
Laptm4a	Human	NM_014713.5	TGAAGCACCTCCTCAGTACG	AGAGCCCTGAATTGCAGCAT	170
Tnf	Human	NM_000594.4	TGGCGTGGAGCTGAGAGATA	TGATGGCAGAGAGGAGGTTG	176
Il6	Human	NM_000600.5	GAGTAGTGAGGAACAAGCCAGA	AAGCTGCGCAGAATGAGATGA	192
Il1b	Human	NM_000576.2	TCGCCAGTGAAATGATGGCT	TGGAAGGAGCACTTCATCTGTT	91
Bax	Human	NM_138763.4	CCCGAGAGGTCTTTTTCCGAG	CCAGCCCATGATGGTTCTGAT	155
Bcl2	Human	NM_000657.3	GGTGGGGTCATGTGTGTGG	CGGTTCAGGTACTCAGTCATCC	89
β-actin	Human	NM_001101.5	CATGTACGTTGCTATCCAGGC	CTCCTTAATGTCACGCACGAT	250

### 2.9 Western Blot

Tissue was lysed in an appropriate amount of RIPA lysis buffer (65 mM Tris-HCl pH 7.5, 150 mM NaCl, 1 mM EDTA, 1% Nonidet P-40, 0.5% sodium deoxycholate, and 0.1% SDS) containing protease inhibitors (0469312001, Roche) and phosphatase inhibitors (4906837001, Roche), followed by ultrasound disruption and centrifugation. The supernatant was collected to obtain total protein. Cells were lysed in SDS lysis buffer (50 mM Tris-HCl pH 6.8, 2% SDS, 10% glycerol) and incubated at 95 °C for 15 minutes, followed by centrifugation to obtain total protein from the supernatant. Protein concentration was determined using a BCA assay kit (23225, Thermo). Equal amounts of protein samples were combined with loading buffer and separated by 10% SDS-PAGE. After electrophoresis, proteins were transferred to a 0.45 μm PVDF membrane (IPVH00010, Millipore). Following transfer, the PVDF membrane was blocked with 5% non-fat milk at room temperature for approximately 1 hour. The PVDF membrane was washed three times with TBST for 5 minutes each, then incubated overnight at 4 °C with the primary antibodies. After washing with TBST, the corresponding species-specific secondary antibody (Jackson ImmunoResearch) was added for 1 hour at room temperature. The proteins were visualized using ECL substrate (1705062, Bio-Rad), and signals were collected using the Bio-Rad Gel Imaging System (ChemiDoc XRS+). The information about the antibodies used is as follows in **Table 2**:

**Table 2:** Information on the antibodies used in the study.

Antibody	Company	Item	Dilution
Laptm4a	Proteintech	30627-1-AP	1:1000
Bax	Abclonal	A19684	1:1000
Bcl2	Abclonal	A20777	1:1000
C-Caspase3	CST	9664	1:1000
Flag	MBL	M185-3L	1:5000
UNC5B	Merck Millipore	ABC89	1:1000
p-AKT	CST	4060	1:1000
AKT	Abclonal	A17909	1:1000
p-mTOR	CST	2971	1:1000
mTOR	Abclonal	A2445	1:1000
p-4E-BP-1	CST	2855	1:1000
4E-BP-1	CST	9644	1:1000
p-S6K	CST	9234	1:1000
S6K	GeneTex	GTX50304	1:1000
β-actin	Abclonal	AC026	1:10000

### 2.10 Statistical analysis

All data are presented as Mean ± SD. Statistical analyses were performed using IBM SPSS Statistics 21.0. For data exhibiting a normal distribution and homogeneity of variance, differences between two groups were assessed using a two-tailed Student’s t-test. For multiple comparisons, a one-way ANOVA was applied, followed by Bonferroni analysis for data meeting the assumption of homogeneity of variance, or Tamhane’s T2 analysis for data demonstrating heteroscedasticity. P<0.05 was considered statistically significant.

## 3 Results

### 3.1 Laptm4a expression significantly increases in renal I/R model

**Figure 1:**
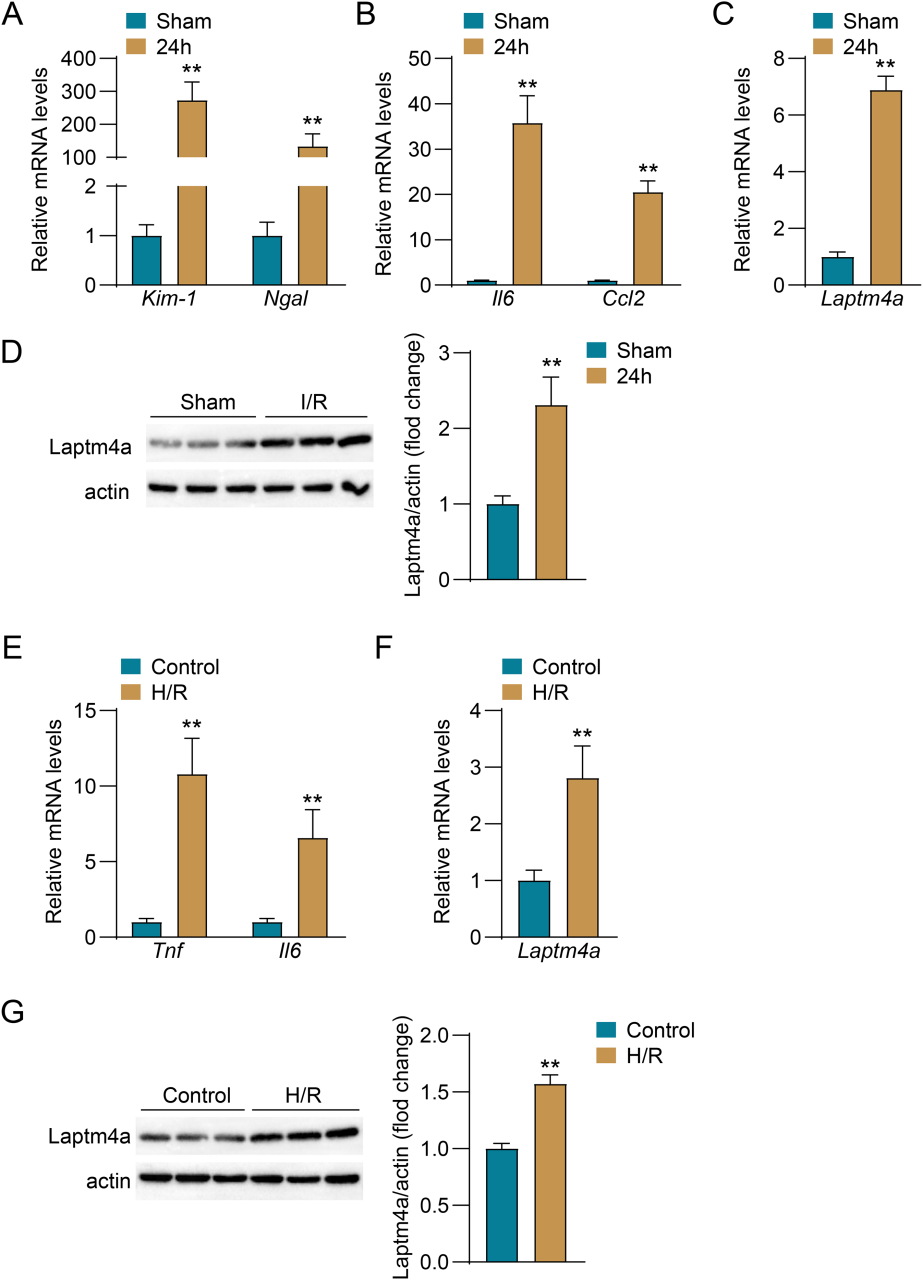
Elevated expression of Laptm4a in the renal ischemia-reperfusion model. **(A)** Analysis of mRNA levels of tubular injury markers Kim-1 and Ngal in the kidney. **(B)** Analysis of mRNA levels of inflammatory response markers Il6 and Ccl2 in the kidney. **(C)** Analysis of mRNA levels of Laptm4a in the kidney. **(D)** Western blot analysis measuring the protein levels of Laptm4a in the kidney. **(E)** Analysis of mRNA levels of inflammatory response markers Tnf and Il6 in HK2cell. **(F)** Analysis of mRNA levels of Laptm4a in HK2 cell. **(G)** Western blot analysis measuring the protein levels of Laptm4a in HK2 cell. All data are represented as the mean ± SD (three independent experiments). **P<0.01.

To investigate the expression of Laptm4a in renal I/R injury, we established a mouse renal I/R model. Compared to sham-operated controls, the mRNA expression of Kim-1 and Ngal (markers of kidney injury) was significantly elevated in murine kidney tissues 24 hours following renal ischemia-reperfusion (I/R) injury (**Figure 1A**). Additionally, the mRNA levels of Il6 and Ccl2 also significantly increased 24 hours after renal ischemia-reperfusion, suggesting that the I/R model was successfully established (**Figure 1B**). The results showed that the mRNA levels of Laptm4a were significantly upregulated after I/R (**Figure 1C**), and Western blot analysis indicated that the protein expression level of Laptm4a also significantly increased after I/R (**Figure 1D**). We also constructed an H/R model in HK2 renal tubular cells *in vitro*.After H/R, the mRNA levels of Tnf and Il6 significantly increased, and the mRNA level of Laptm4a also significantly increased (**Figures 1E, F**); Western blot results showed that the protein expression level of Laptm4a significantly increased after H/R (**Figure 1G**). These results suggest that Laptm4a is involved in renal ischemia-reperfusion injury.

### 3.2 Laptm4a deficiency alleviates renal ischemia-reperfusion injury

**Figure 2:**
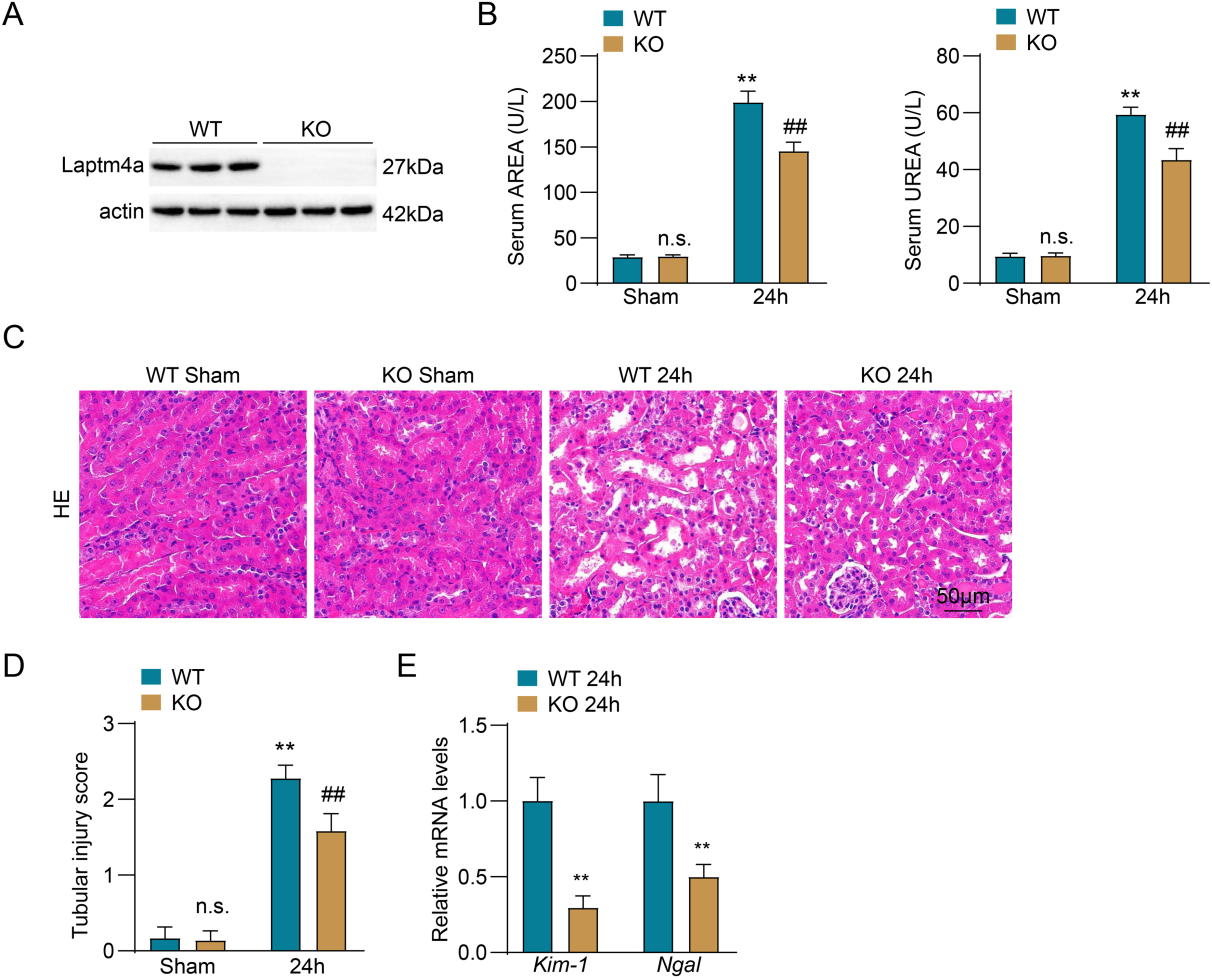
Laptm4a deficiency alleviates renal ischemia-reperfusion injury. **(A)** Western blot analysis measuring the levels of Laptm4a protein. **(B)** Analysis of serum levels of serum AREA and UREA in mice. **(C)** Respresent HE staining **(C)** images and corresponding quantitative analysis **(D)** result of renal tissue. Bar=50μm. **(E)** Analysis of mRNA levels of tubular injury markers Kim-1 and Ngal in the kidney. All data are represented as the mean ± SD(three independent experiments). n.s., not significant; **p < 0.01 versus respective Sham-operated group. ##p < 0.01 versus WT-IRI(24h) group.

To further investigate the role of Laptm4a in renal ischemia-reperfusion injury, we used Laptm4a gene knockout mice (**Figure 2A**) to establish a renal IRI model. Serum creatinine(Cr) and urea nitrogen(BUN) are key indicators for evaluating renal function. As shown in **Figures 2B–D**, there were no obvious differences in renal function or renal tissue morphology(HE staining) between Laptm4a-KO mice and wild-type (WT) mice in the sham group., And elevated renal function markers, specifically BUN and Cr levels, along with evident renal pathological damage in mice following ischemia-reperfusion, indicate the successful establishment of the mouse ischemia-reperfusion injury model. Compared to the WT mice in I/R group, renal function and tissue damage induced by I/R was significantly reduced following Laptm4a knockout (**Figures 2B–D**). Kim-1 and Ngal are key molecules for tubular injury, and the results showed that Laptm4a knockout significantly reduced the mRNA levels of I/R-induced Kim-1 and Ngal (**Figure 2E**), indicating that renal tubular injury was alleviated.

### 3.3 Laptm4a deficiency reduces inflammatory response and cell apoptosis after renal I/R

**Figure 3:**
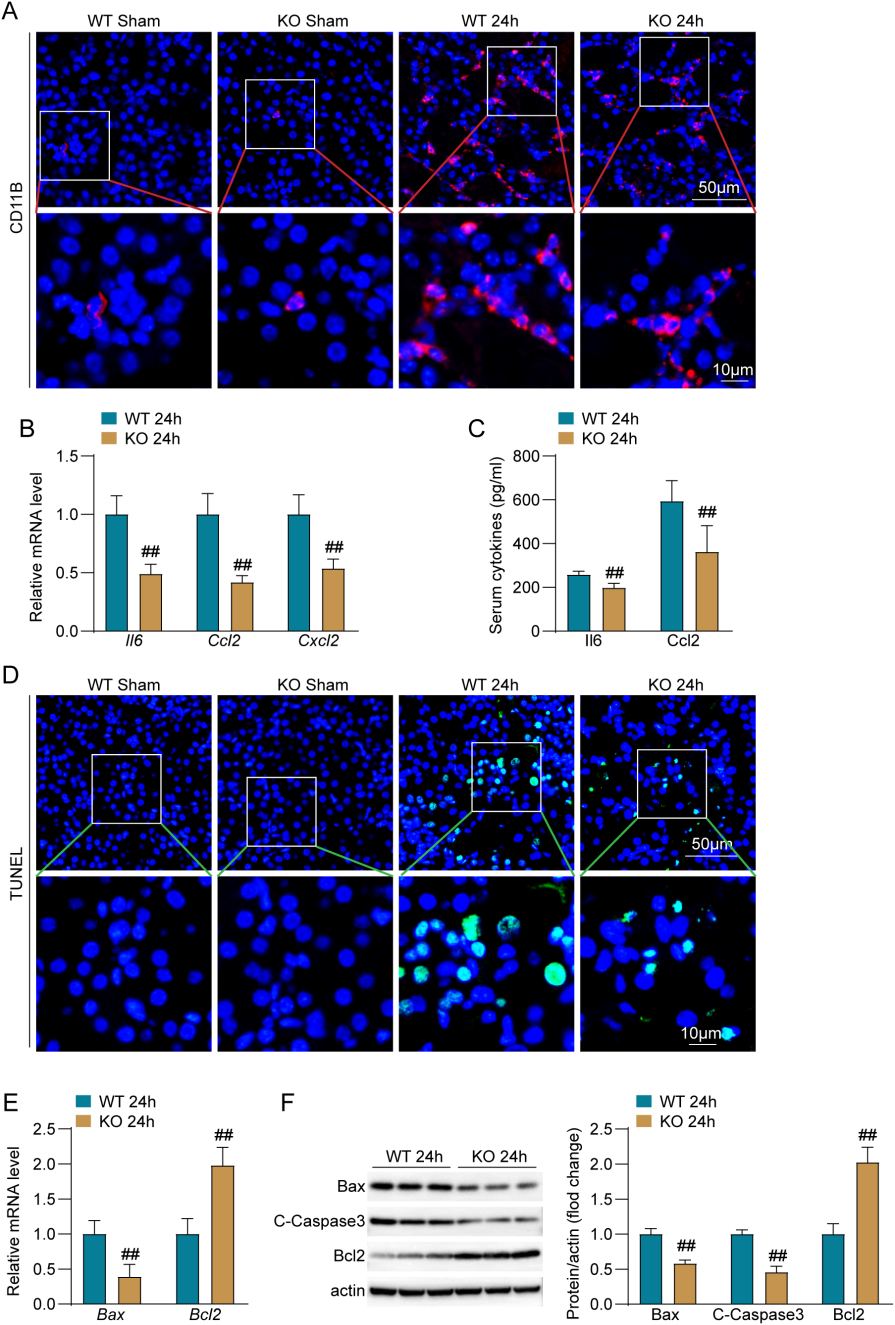
Laptm4a deficiency alleviates inflammation and apoptosis after renal I/R. **(A)** Immunofluorescence staining of CD11b-positive inflammatory cells (red) in kidney of mice. Bar=50μm,10μm(zoom). **(B)** Analysis of mRNA levels of inflammatory response markers Il6, Ccl2, and Cxcl2 in the kidney. **(C)** Analysis of serum levels of cytokines Il6 and Ccl2 in mice. **(D)** Representative images of TUNEL staining (20×) (green) in kidney.Bar=50μm, 10μm(zoom). **(E)** Analysis of mRNA levels of apoptotic markers Bax and Bcl2 in the kidney. **(F)** Western blot analysis of the expression of Bax, C-Caspase3, and Bcl2 in the kidney.All data are represented as the mean ± SD (three independent experiments). ##P<0.01.

The inflammatory response and apoptosis play critical roles in renal IRI (1). Therefore, this study investigates the impact of Laotm4a on these processes during renal IRI. Initially, we conducted CD11b immunofluorescence staining to assess the impact of Laptm4a on the infiltration of inflammatory cells. Compared to the sham operation group, the number of CD11b-positive inflammatory cells (red) significantly increased in the kidney sections of wild-type mice 24 hours after I/R. Laptm4a knockout significantly reduced the CD11b-positive inflammatory cells (**Figure 3A**). Subsequently, we investigated the production of inflammatory mediators following renal IRI. Compared to wild-type mice, the mRNA levels of inflammatory markers Il6, Ccl2, and Cxcl2 were significantly lower in the Laptm4a-KO group 24 hours after I/R (**Figure 3B**), the serum concentrations of the cytokines IL-6 and CCL2 were significantly decreased (**Figure 3C**). Furthermore, we conducted TUNEL staining to elucidate the presence of apoptosis. Compared to the sham operation group, TUNEL-positive apoptotic cells (green) significantly increased in the kidney sections of wild-type mice 24 hours after I/R. Laptm4a knockout significantly reduced the TUNEL-positive apoptotic cells (green) after I/R (**Figure 3D**). qRT-PCR and Western blot analysis were employed to assess the expression levels of apoptosis-related molecules, specifically Bax, Bcl-2, and cleaved Caspase-3, in kidney tissues. The findings indicated a significant reduction in the mRNA expression level of the pro-apoptotic molecule Bax, alongside a significant increase in the mRNA expression level of the anti-apoptotic molecule Bcl2, in the Laptm4a-KO group of mice compared to the wild-type mice, 24 hours post-ischemia/reperfusion (I/R) (**Figure 3E**). Western blot analysis showed that, compared to wild-type I/R mice, the expression levels of the pro-apoptotic molecules Bax and C-Caspase3 were significantly lower in Laptm4a-KO I/R mice, while the expression of the anti-apoptotic molecule Bcl2 was significantly higher (**Figure 3F**). Taken together, these results demonstrated that Laptm4a deficiency relieves inflammation and cell apoptosis after renal IRI.

### 3.4 Silencing Laptm4a reduces cell injury, inflammatory response and apoptosis in HK2 After H/R

**Figure 4:**
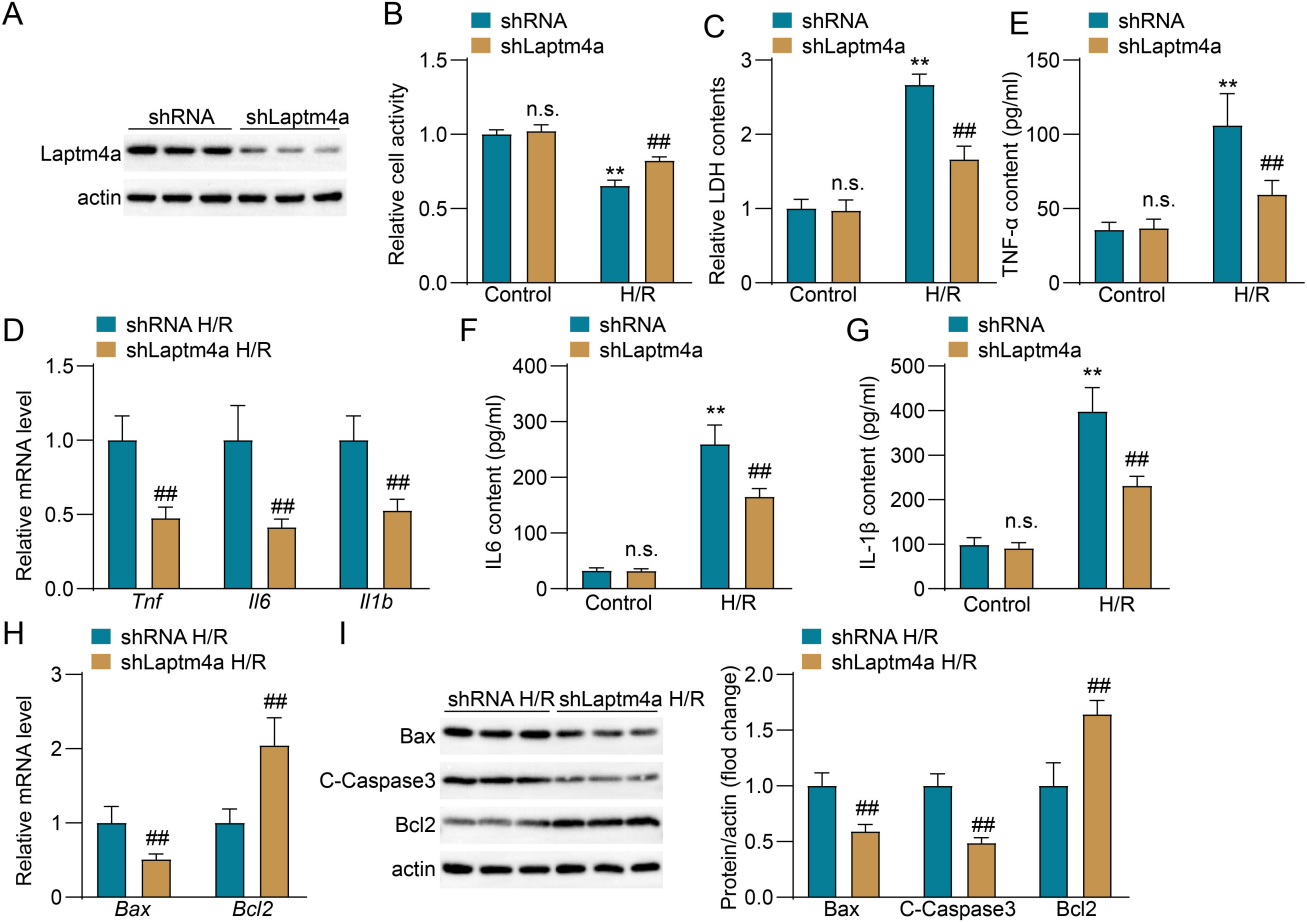
Silencing Laptm4a alleviates cell damage, inflammation and apoptosis in human renal tubular epithelial cells (HK2) after H/R. **(A)** Western blot analysis measuring Laptm4a protein levels in shRNA and shLaptm4a HK2 cell. **(B)** Cell viability assessment and **(C)** LDH level assessment in shRNA and shLaptm4a HK2 cell. **(D)** mRNA level detection of Tnf, IL6, and IL1b in shRNA and shLaptm4a HK2 cell. **(E–G)** TNF-α, IL6 and IL-1β detection in shRNA and shLaptm4a HK2 cell. **(H)** mRNA level detection of Bax and Bcl2 in shRNA and shLaptm4a HK2 cell. **(I)** Western blot analysis of the expression of Bax, C-Caspase3, and Bcl2 in shRNA and shLaptm4a HK2 cell. All data are represented as the mean ± SD (three independent experiments). n.s., not significant; **p < 0.01 versus respective Control group; ##p < 0.01 versus shRNA-H/R group.

Renal tubular cell injury plays a critical role in the pathophysiology of renal IRI (2); therefore, we conducted a further investigation into the impact of Laptm4a on renal tubular cell injury. The classical H/R model was employed to simulate ischemia-reperfusion injury *in vitro*. Western blot analysis showed that the protein level of Laptm4a was significantly reduced in HK2 cells after silencing Laptm4a,indicating the successful construction of cell line models with Laptm4a knockdown (**Figure 4A**). After H/R, the cell viability in the shLaptm4a group was significantly higher than that in the shRNA group, while no significant difference in cell viability was observed in the control group (**Figure 4B**). Following H/R, the LDH contents were significantly lower in the shLaptm4a group compared to the shRNA group, suggesting that silencing Laptm4a can significantly reduce cell damage, with no significant difference in LDH levels observed in the control group (**Figure 4C**). The mRNA expression levels of inflammatory factors (Tnf, IL6, and IL-1β) within the cells of the shLaptm4a H/R group (**Figure 4D**), as well as the concentrations of these inflammatory factors (Tnf, IL6, and IL-1β) (**Figures 4E–G**) in the culture medium, were reduced compared to those observed in the shRNA H/R group (**Figures 4D–G**). Meanwhile, Laptm4a knockdown in HK2 decreased proapoptosis-associated molecular (BAX and C-caspase 3) expression and promoted BCL-2 expression after H/R treatment compared to the same expressions in the control group(**Figure 4H**). WB showed similar results in **Figure 4I**. Collectively, these findings clearly demonstrated that Laptm4a knockdown suppresses H/R-induced inflammation and apoptosis in HK2 cells.

### 3.5 Overexpression of Laptm4a promotes cell injury, inflammation and apoptosis in human renal tubular epithelial cells (HK2) After H/R

**Figure 5:**
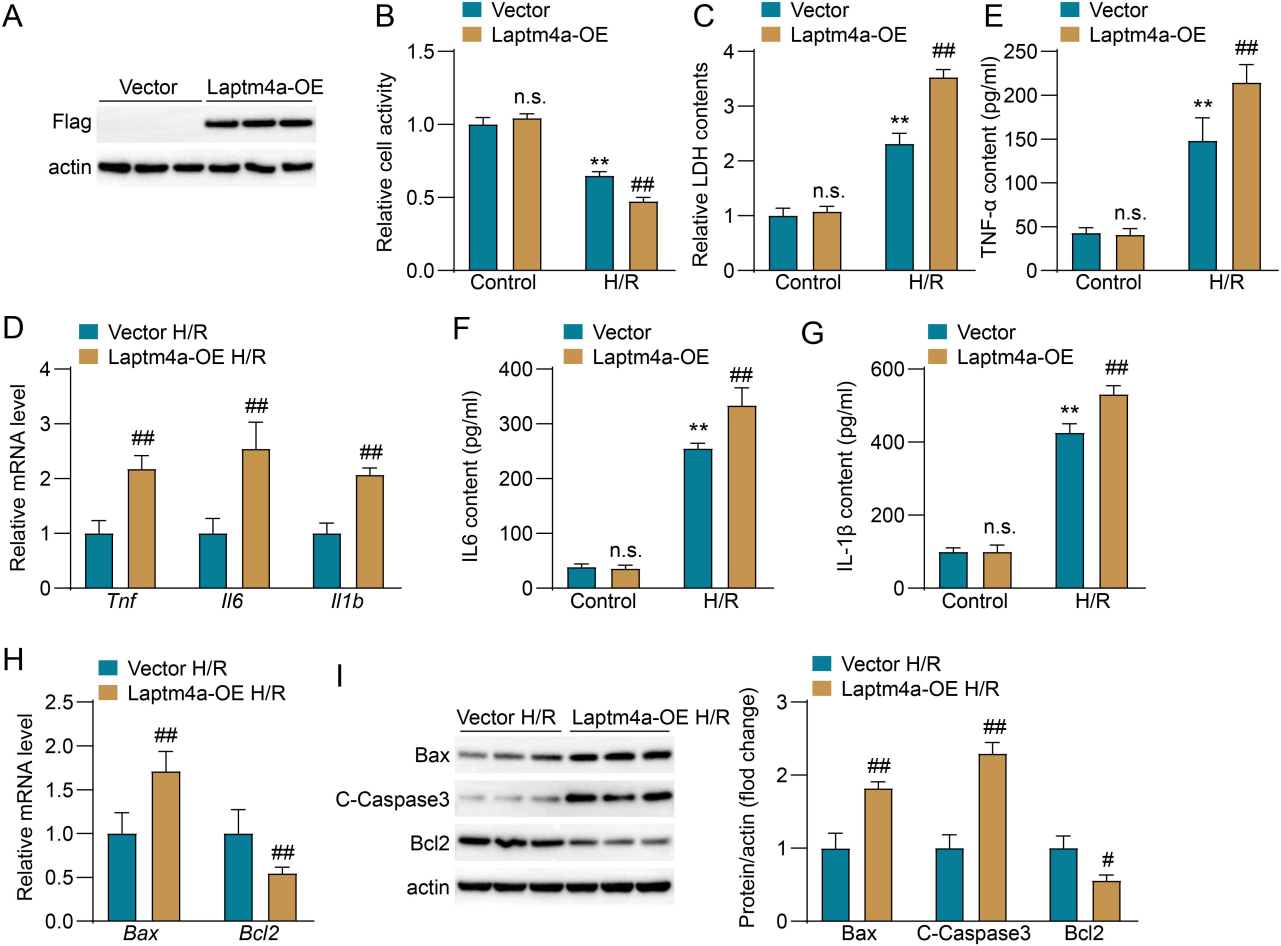
Overexpression of Laptm4a promotes cell damage, inflammation and apoptosis in human renal tubular epithelial cells (HK2) after H/R. **(A)** Measurement of Flag protein levels in Vector and Laptm4a-OE HK2 cells using Western blotting. **(B)** Cell viability assessment and **(C)** LDH level assessment of Vector and Laptm4a-OE HK2 cell indifferent groups. **(D)** Measurement of mRNA levels of Tnf, IL6, and IL1b in HK2 cell. **(E–G)** Detection of TNF-α,IL6 and IL-1β levels in HK2 cell. **(H)** Measurement of mRNA levels of Bax and Bcl2 in HK2 cell. **(I)** Western blot analysis of Bax, C-Caspase3, and Bcl2 expression in HK2 cell.All data are represented as the mean ± SD(three independent experiments). n.s., not significant; **p < 0.01 versus respective Control group; ##p < 0.01 versus Vector-H/R group.

To further confirm the role of Laptm4a in HK2 cells during H/R, we constructed a Laptm4a overexpression cellline (Laptm4a-OE). The results showed that compared to the control group (Vector), the expression of Flag-Laptm4a was significantly increased in the Laptm4a-OE group, indicating that the overexpression cell line was successful established (**Figure 5A**). After H/R, the Laptm4a-OE group exhibited lower cell viability (**Figure 5B**) and higher LDH levels (**Figure 5C**) than the Vector group, with no differences in the control group. Inflammatory markers Tnfα, IL6, and IL-1β (**Figures 5D–G**) were significantly elevated in the Laptm4a-OE group, indicating increased inflammation. Apoptosis markers showed higher Bax and C-Caspase3 levels and lower Bcl2 levels in the Laptm4a-OE group compared to the Vector group(**Figures 5H, I**). Similar results in WB was showed in **Figure 5I**. These findings suggest that Laptm4a overexpression enhances cell apoptosis and inflammation *in vitro*.

### 2.6 Laptm4a negative regulate the activation of UNC5B-AKT-mTOR signaling pathway

**Figure 6:**
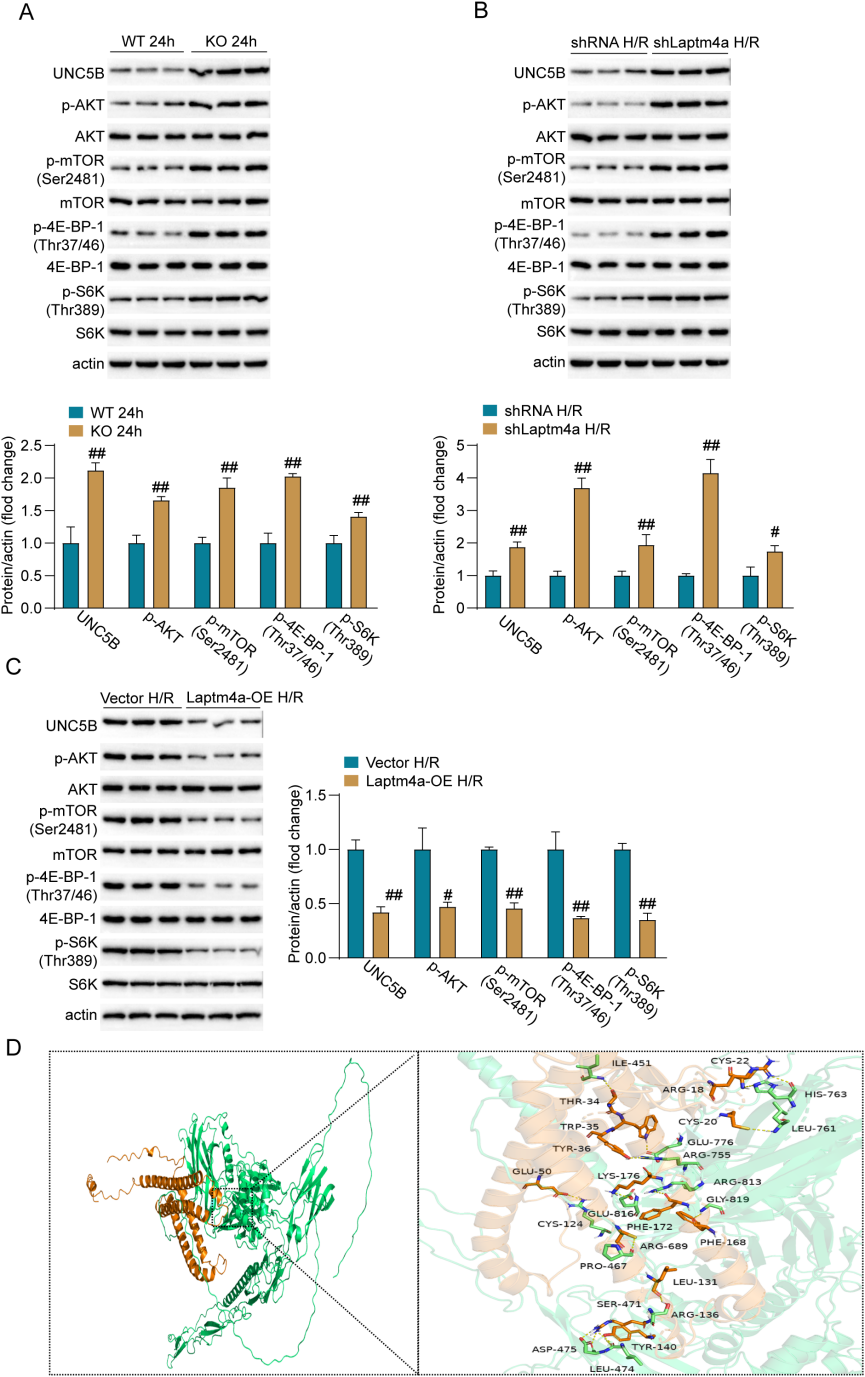
Laptm4a inhibits the UNC5B-AKT-mTOR signaling pathway. **(A)** Protein levels of UNC5B, p-AKT, p-mTOR, p-4E-BP-1, and p-S6K in wild-type and Laptm4a-KO mice after 24 hours of I/R. **(B)** Protein levels of UNC5B, p-AKT, p-mTOR, p-4E-BP-1, and p-S6K in shRNA and shLaptm4a HK2 cells after H/R. **(C)** Protein levels of UNC5B, p-AKT, p-mTOR, p-4E-BP-1, and p-S6K in Vector and Laptm4a-OE HK2 cells after H/R. **(D)** Structure-based protein interaction interface analysis between LAPTM4A and UNC5B. Images depict the predicted LAPTM4A-UNC5B complex structure, with interaction hotspot residues labeled.All data are represented as the mean ± SD (three independent experiments). ##P<0.01.

To explore how Laptm4a influences Renal IRI, we used Flag antibody to immunoprecipitate lysates from H/R-stimulated Laptm4a-overexpressing HK2 cells and analyzed the proteins for UNC5B via mass spectrometry. UNC5B deletion is known to worsen tissue damage in acute kidney injury (10) and significantly promotes the activation of the AKT-mTOR signaling axis (11). Then, we assessed the impact of changing Laptm4a expression on the UNC5B-AKT-mTOR signaling axis using Western blot. Western blot analysis showed that in animal experiments, compared to wild-type mice, the kidney levels of UNC5B, p-AKT, p-mTOR, p-4E-BP-1, and p-S6K were significantly increased in Laptm4a knockout mice (**Figure 6A**). In cellular experimental models, compared to the shRNA group, the levels of UNC5B, p-AKT, p-mTOR, p-4E-BP-1, and p-S6K were significantly higher in the shLaptm4a group (**Figure 6B**). In the overexpression experiments, compared to the Vector group, the levels of UNC5B, p-AKT, p-mTOR, p-4E-BP-1, and p-S6K were significantly reduced in the Laptm4a-OE group (**Figure 6C**). The interaction mechanism between LAPTM4A and UNC5B was subsequently elucidated through molecular docking studies. Three-dimensional protein structure files for LAPTM4A and UNC5B were obtained from the RCSB Protein Data Bank (https://www.rcsb.org/). Comprehensive three-dimensional and two-dimensional force analyses and visualizations were conducted using Discovery Studio and Ligplus software. The analyses revealed a binding energy of −52.4 kcal/mol between LAPTM4A and UNC5B. In the two-dimensional representation, green dashed lines indicate hydrogen bonds, while red dashed lines signify hydrophobic interactions, as shown in **Supplementary Figure 1**. Generally, a more negative binding energy value suggests enhanced binding stability between a ligand and its target protein. In the three-dimensional visualization, the LAPTM4A protein is depicted as a gold ribbon structure, whereas the UNC5B protein is represented by a green ribbon structure, as illustrated in **Figure 6D**. These results suggest that Laptm4a mediates renal ischemia-reperfusion injury by inhibiting the UNC5B-AKT-mTOR signaling pathway.

### 2.7 Laptm4a modulates renal IRI progression via the UNC5B-AKT-mTOR pathway

**Figure 7:**
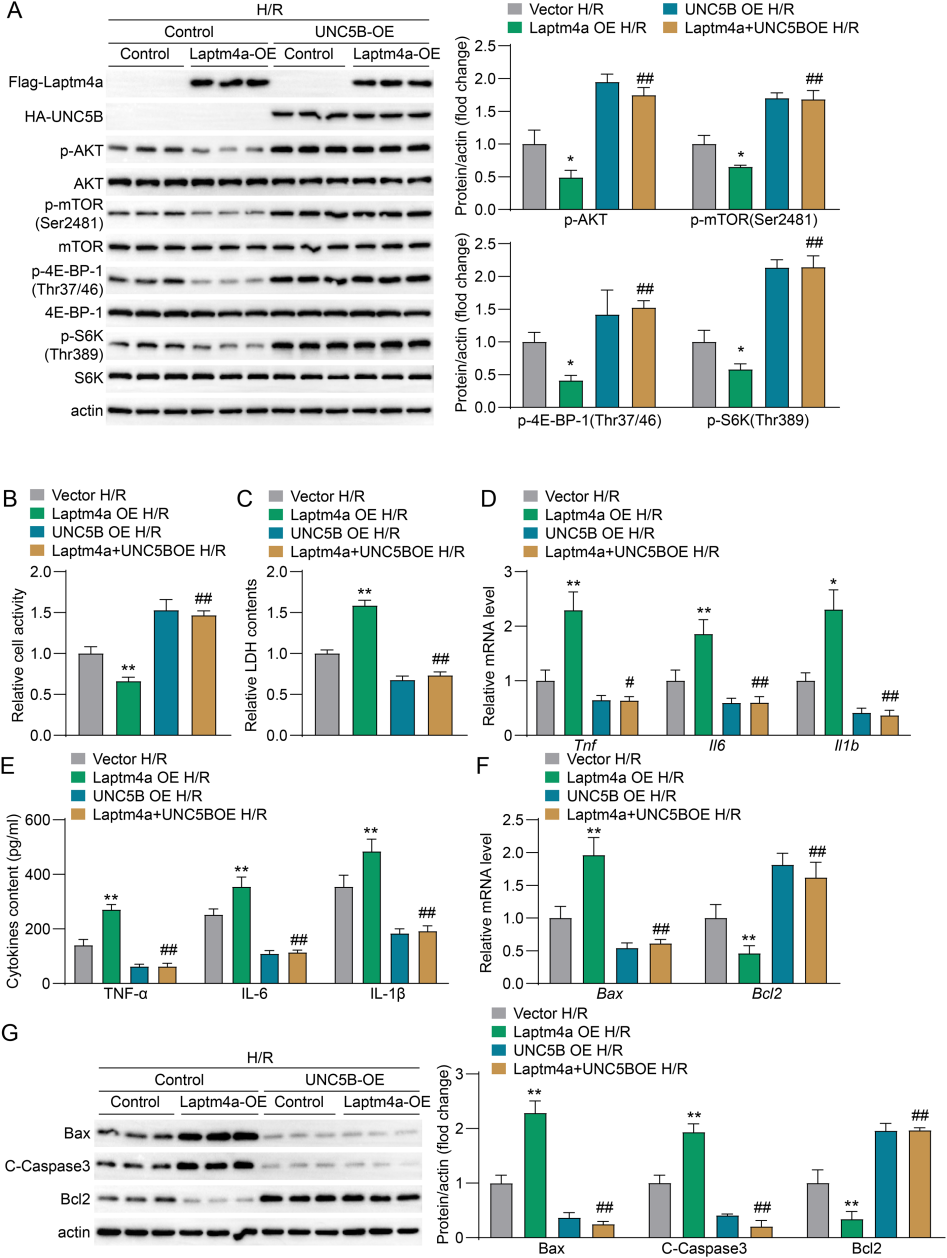
The effect of Laptm4a on H/R injury in renal tubular epithelial cells (HK2) is mediated by UNC5B activity. **(A)** Measurement of protein levels and phosphorylation status of Laptm4a, UNC5B, AKT, mTOR, 4E-BP-1, and S6K in each group. **(B)** Assessment of cell viability levels in each group. **(C)** Detection of LDH levels in each group. **(D)** Measurement of mRNA levels of Tnf, Il6, and Il1β in each group. **(E)** Detection of cytokine levels TNF-α, IL-6, and IL-1β in each group; **(F)** Measurement of mRNA levels of apoptosis markers Bax and Bcl2 in each group; **(G)** Western blot analysis of apoptotic molecules Bax, C-Caspase3, and Bcl2 expression in each group.All data are represented as the mean ± SD(three independent experiments). n.s., not significant; *P<0.05; **P<0.01; #P<0.05; ##P<0.01.

To further validate the role of UNC5B in mediating Laptm4a’s effects on H/R injury in HK2 cells, we designed a rescue experiment. The results showed that compared to the Vector group, the expression levels of p-AKT, p-mTOR, p-4E-BP-1, and p-S6K were significantly reduced in the Laptm4a overexpression group, and this decrease was rescued upon overexpression of UNC5B (**Figure 7A**). The assessment of cell viability and LDH levels after H/R treatment indicated that the group with Laptm4a overexpression exhibited a marked reduction in cell viability and a significant elevation in LDH release compared to the Vector group. Whereas, the overexpression of UNC5B mitigated the decline in cellular activity and the increase in LDH release induced by LAPTM4A overexpression, effectively restoring cell viability and ameliorating LDH levels (**Figures 7B, C**). At the level of inflammatory factor content and mRNA expression after H/R treatment, the concentrations of TNF, IL-6, and IL-1β were significantly elevated in the LAPTM4A overexpression group compared to the Vector group (**Figure 7D**). Furthermore, the overexpression of UNC5B alleviated the effects of Laptm4a overexpression on the increase of cytokines (**Figure 7E**). The overexpression of UNC5B significantly mitigated the increase in apoptosis induced by Laptm4a overexpression in HK2 cells, as confirmed by qPCR and western blotting (**Figures 7F, G**). Taken together, these findings indicate that UNC5B activity is crucial for the role of increased Laptm4a expression in aggravating renal IRI.

## 4 Discussion

Renal IRI is a multifaceted condition characterized by various etiological factors that significantly impact the prognosis of kidney transplantation. IRI influences all areas of the nephron, including the tubules, glomerulus, interstitium, and vasculature, yet the proximal tubular cells are the most commonly affected. In the present study, we identified lysosomal-associated protein transmembrane 4A (Lapm4a) as a promising therapeutic target for Renal IRI. Our findings indicate that Laptm4a exacerbates inflammation and apoptosis by inhibiting the UNC5B-PI3K-AKT signaling pathway. Although lysosomal proteins have been recognized as therapeutic targets for various diseases (12–14), particularly cancer gliomas (15–17), their role in renal IRI injury warrants further investigation. This study systematically reveals the key role of Laptm4a in renal ischemia-reperfusion injury (IRI) for the first time. Our present study elucidates the regulatory function of Laptm4a in I/R-induced renal injury, thereby underscoring its potential as a therapeutic target for the amelioration of such injuries.

The primary causes and clinical manifestations of renal IRI are apoptosis and the inflammatory response. Previous research on Laptm4a has predominantly concentrated on its regulatory role in tumors and glioma, as well as the associated mechanisms. It has been established that the upregulation of Laptm4a promotes M2 polarization of tumor-associated macrophages (TAMs) and facilitates glioma progression by enhancing cell proliferation and invasion (17). Laptm4a is localized to the lysosome and can co-immunoprecipitated with, and be ubiquitinated by, the E3 ubiquitin ligase Nedd4, playing a role in the membrane sorting of LAPTM4 proteins (18). The core finding of this study is the identification of Laptm4a as a negative regulator of UNC5B protein levels, inhibiting AKT and downstream signaling, leading to increased apoptosis and inflammation. While the exact biochemical mechanism requires further elucidation, we propose several plausible hypotheses grounded in the biology of lysosomal membrane proteins. Laptm4a may facilitate the lysosomal degradation of UNC5B, act as an adaptor to promote its ubiquitination [as seen with other LAPTM family members (18, 19)], or otherwise sequester UNC5b to promote its turnover. Delineating the exact nature of this interaction represents a key direction for future research.

In the regulatory pathways of renal ischemia, the AKT-mTOR signaling pathway plays an important role. Existing studies have shown that the AKT-mTOR pathway has a dual regulatory effect in renal IRI: on one hand, it can promote cell survival and proliferation (20); on the other hand, it may exacerbate the degree of inflammatory responses and apoptosis (21, 22) by activating downstream pathways. Specifically, activation of AKT can regulate the expression of various inflammatory factors and alter the activity of apoptosis pathways, thereby playing a central role in the interaction between inflammation and apoptosis. The mechanisms revealed in this study may also be applicable to other organs, as the PI3K-AKT pathway plays a similarly core protective role in the IRI of the heart, liver, and other organs (23). Our study demonstrated that both over-expression and knockdown of Laptm4a significantly modulated the AKT-mTOR signaling pathway, a pathway previously established as critical in the context of acute kidney injury (AKI). Xu et al. (24) found that schisandrin B activates AKT1 to reduce apoptosis and oxidative stress, mitigating renal ischemia-reperfusion injury. Xu et al. (25) demonstrated that IL-22 specifically targets the proximal renal tubular epithelium, leading to the activation of STAT3 and AKT pathways, inhibition of apoptosis, and subsequent mitigation of renal ischemia-reperfusion injury. ADAMTS13 enhances NO production via AKT and eNOS activation, improving endothelial function and reducing inflammation and apoptosis in ischemic kidneys (26). Lieberthal et al. (27) showed that AMPK-mediated Akt activation protects tubular cells from stress-induced apoptosis in ischemic AKI. Li et al. (28) discovered that tubular β-chain protein activates AKT/p53 and FOXO3/PGC-1α pathways, reducing mitochondrial dysfunction and cell death in acute kidney injury. Song et al. demonstrated that Salvianolic acid A has the potential to ameliorate renal ischemia-reperfusion (I/R) injury and enhance tubular cell survival by activating the Akt/mTOR/4EBP1 signaling pathway. These findings align with the results presented in our study, which showed that the knockdown of Lampt4a enhances the phosphorylation of Akt, mTOR, and 4EBP1, thereby mitigating renal injury in the context of renal I/R injury (29).

Research on UNC5B in acute kidney injury (AKI) shows that UNC5B deficiency worsens kidney damage. In renal ischemia-reperfusion and cisplatin-induced AKI models, Ranganathan et al. (10) found that UNC5B−/flox/GGT-cre mice had more severe kidney injury and dysfunction than UNC5B−/flox and wild-type mice, with increased tubular apoptosis and inflammation. UNC5B activates the Akt pathway, suppressing p53 and reducing apoptosis and inflammation. Similarly, Cao et al. (30) showed that UNC5B antisense RNA 1 (UASR1) activates the AKT and mTOR pathways, promoting renal cell proliferation and repair. In ovarian studies, Shen et al. (31) found that Jiawei Qingxin Zishen Decoction (JWQZD) reduces granulosa cell apoptosis and boosts ovarian reserve by activating the Netrin/UNC5B and AKT pathways. These findings are consistent with the UNC5B/AKT signaling pathway’s suppression of inflammation and apoptosis demonstrated in this study.

LAPTM4A is a 27 kDa transmembrane protein, initially identified as mouse transport protein (MTP) (32). Studies (17)show that knocking down LAPTM4A in BV2 microglia shifts macrophages from M2 to M1, increasing pro-inflammatory cytokines like IL-6 and TNF-α. In gestational diabetes, high glucose activates the hsa_circ_0042260/miR-4782-3p/LAPTM4A axis, raising LAPTM4A levels, which leads to greater apoptosis and inflammation in HK-2 (33). Despite limited research on LAPTM4A in renal contexts, we found that its relative, LAPTM5, promotes renal tubular aging and secretes SASP factors (e.g., IL-6, TNF-α, CTGF) via the WWP2/Notch1 signaling pathway, worsening renal injury in chronic kidney disease (34). Previous studies show that LAPTM5 enhances proinflammatory signaling in macrophages. LAPTM5 deficiency in mice or knockdown in RAW264.7 cells reduces TNF-receptor-mediated NF-κB and MAPK pathway activation (35). In hematopoietic cells, LAPTM5 recruits E3 ubiquitin ligases for lysosomal degradation of targets, inhibiting AKT phosphorylation (19). Both LAPTM5 and LAPTM4A inhibit AKT, but LAPTM5 does so via the classical ubiquitin-mediated degradation pathway, while LAPTM4A interacts with the receptor UNC5B. This illustrates the functional diversity within the LAPTM family and highlights LAPTM4A’s unique role in organ injury through a new regulatory mechanism. Previous research has shown LAPTM4A is widely expressed in various kidney tubular segments (7, 36). In this study, we found increased LAPTM4A expression in renal tubular epithelial cells after renal IRI, but its expression in other renal cell types and potential regulatory effects remain unclear, warranting further research.

This study also has some limitations. An important limitation of our study is the exclusive use of male mice. This choice was initially made to minimize experimental variability, particularly given the potential confounding effects of the estrous cycle on ischemia-reperfusion injury susceptibility in females. However, we acknowledge that this precludes any conclusions regarding the role of Laptm4a in female subjects. Future studies are unequivocally required to include female animal models to determine whether the pathogenic function of Laptm4a and the activity of the Laptm4a-UNC5B-AKT/mTOR axis are sex-dependent. Elucidating potential sex differences will be crucial for a comprehensive understanding of Laptm4a’s role in AKI and for informing any future translational strategies. Additionally, while we confirmed that Laptm4a acts through the UNC5B-PI3K-AKT axis, the precise regulatory mechanism between the two (such as whether direct binding or phosphorylation modifications exist) requires further investigation. Furthermore, this study lacks verification from tissue samples of human AKI patients, which prevents us from confirming the clinical correlation between Laptm4a expression levels and disease severity or prognosis. Although appropriate controls were set up, validation through conditional knockout models or small molecule inhibitors is still needed. We utilized Laptm4a knockout models to elucidate the impact of Laptm4a deletion on renal IRI *in vivo*. *In vitro*, we investigated the regulatory role of Laptm4a on HK2 cells. It is important to consider the limitations of our *in vivo* model. The use of a global Laptm4a knockout mouse means we cannot formally rule out potential contributions from systemic factors or Laptm4a deficiency in other cell types (e.g., immune cells) to the observed protective phenotype. However, the consistent amelioration of injury across multiple parameters (function, histology, inflammation, apoptosis) in global KO mice, coupled with our *in vitro* findings that specific knockdown of Laptm4a in renal tubular epithelial (HK2) cells was sufficient to recapitulate the protective effects (reduced LDH release, enhanced viability, suppressed inflammation and apoptosis), strongly supports a kidney-intrinsic, and likely a tubular epithelial-intrinsic, role for Laptm4a in mediating IRI. Nonetheless, we did not conduct functional validation of Laptm4a over-expression *in vivo*, nor did we explore the effects of renal tubular cell-specific Laptm4a overexpression on renal IRI. Another limitation lies in the fact that, in animals and cells, it remains unclear what distinct effects complete knockout versus partial knockout would produce. Future studies employing tubule-specific conditional knockout and over-expression models would be valuable to definitively confirm this cell-autonomous mechanism. These limitations provide directions for future research, including conducting multi-center clinical sample analyses and developing specific targeting tools.

In summary, the present study accomplishes two key advances. First, it identifies the lysosomal membrane protein Laptm4a as a novel and potent positive mediator of renal IRI. Second, and more significantly, it reveals an entirely new regulatory axis—Laptm4a-UNC5B-AKT/mTOR—that is reported for the first time in the context of kidney injury. This discovery is conceptually innovative because it establishes a previously unrecognized functional bridge between a lysosomal transmembrane protein and the classical pro-survival/metabolic AKT/mTOR signaling pathway, with the guidance receptor UNC5B serving as the critical linchpin. By delineating this novel Laptm4a-UNC5B interface, our work not only deepens the understanding of the molecular underpinnings of AKI but also unveils a promising and unexplored target for therapeutic intervention.

## Data Availability

The raw data supporting the conclusions of this article will be made available by the authors, without undue reservation.
