# Supplementary material for: Laptm4a mediates renal ischemia-reperfusion injury by regulating the UNC5B-AKT/mTOR signaling pathway
Source: Front Immunol. 2026 Mar 24;17:1683343. doi: 10.3389/fimmu.2026.1683343 (PMC13053316; doi:10.3389/fimmu.2026.1683343)
Supplement: Supplementary file 1 [file DataSheet1.doc]

Supplementary Figure 1. Analysis and visualization of two-dimensional angles of action forces between LAPTM4A protein and UNC5B protein.

The 816th GLU amino acid residue of the UNC5B protein can bind to the 176th LYS amino acid residue of the LAPMT4A protein through a hydrogen bonds with lengths of 2.81Å; the 471th Ser

amino acid residue of the UNC5B protein can bind to the 131th LEU amino acid residue of the LAPTM4A protein through a hydrogen bonds with lengths of 3.01Å. In the two-dimensional diagram, green dashed lines represent hydrogen bonds and red dashed lines represent hydrophobic interactions.
